# Genomic characterization of vancomycin-resistant enterococci in Norwegian poultry

**DOI:** 10.1371/journal.pone.0324789

**Published:** 2025-06-04

**Authors:** Rikki Franklin Frederiksen, Anne Margrete Urdahl, Jannice Schau Slettemeås, Silje Granstad, Roger Simm, Karin Lagesen

**Affiliations:** 1 Department of Animal Health and Food Safety, Norwegian Veterinary Institute, Ås, Norway; 2 Department of Biosciences, University of Oslo, Oslo, Norway; Yale University School of Medicine, UNITED STATES OF AMERICA

## Abstract

Vancomycin resistant enterococci (VRE) are a globally leading cause of nosocomial infections in humans, and a potential animal VRE reservoir, especially in poultry, is of concern. It has been suggested that the presence of VRE in broilers has been prolonged due to co-selection of narasin resistance (*narAB* genes) and vancomycin resistance (*vanA* genes), and that these genes may be present on the same plasmids. The aim of this study was to use whole genome sequencing to characterize and compare both the chromosomes and plasmids of VRE isolates from poultry in Norway, and to elucidate whether co-localisation of *narAB* and *vanA* genes on the same genetic elements, or clonal spread, could explain the persistence of VRE. A total of 30 VRE isolates from the years 2002–2013 were included, 23 from broiler flocks and seven from turkey flocks. WGS analyses showed that the isolates were genetically diverse with the number of SNPs ranging from 10 to 2807. The isolates belonged to 15 different sequence types, though all carried a plasmid similar to pVEF plasmids, and contained both *vanA* and *narAB.* A comparative gene analysis indicated that *narAB* is carried on a composite transposon, and that interspecies transfer of the plasmid between *Enterococcus faecium* and *Enterococcus hirae* may occur. The absence of transfer genes on the pVEF-like plasmids and their presence on a megaplasmid suggest that the megaplasmid probably act as a helper plasmid. Overall, the results support that the use of narasin in broilers may be a risk factor for a persistent reservoir of VRE in broilers.

## Introduction

Enterococci are commensals of the gastrointestinal tract of animals and humans. Vancomycin resistant enterococci (VRE) of the species *Enterococcus faecalis* and *Enterococcus faecium* are important causes of healthcare associated infections, especially in immunocompromised individuals [1–3]. As a result, vancomycin resistant *E. faecium* were included on the WHO global priority pathogen list [4]. VRE are also found in animal enterococci reservoirs [5,6], but it has been suggested that VRE of animal origin are rare among hospital infections [7], and that opportunistic enterococci are host-specific [5,6,8]. Still a potentially large animal reservoir is of concern as VRE from broilers have been shown to cause transient colonization of broiler farmers [9,10]. Furthermore, transfer of vancomycin resistance between isolates of animal and human origin has been demonstrated both *in vitro* and *in vivo* in animals and humans [11–14]. In addition, inter-species transfer of vancomycin resistance between VRE and other pathogens, such as methicillin resistant *Staphylococcus aureus* (MRSA) has been shown to occur [15,16].

In Europe and Norway, all VRE isolated from broilers have been *E. faecium* and have proven to carry the *vanA* gene cluster [9,17–29]. VRE emerged in broiler populations in many European countries due to cross-resistance to the previously used growth promoting glycopeptide feed additive avoparcin [30–34]. Despite the ban of avoparcin in Norway in 1995 and a non-existing use of vancomycin, VRE could still be isolated from Norwegian broilers using selective cultivation up until 2014. Between 2015 and 2024, the national surveillance program conducted VRE monitoring in 2018, 2020, 2022 and 2024, during which VRE was not detected [19,35–37]. The reason for the prolonged persistence of VRE up until at least 2014, is unknown, though it has been suggested to be connected to plasmid maintenance systems and unknown selection pressure [18].

An unknown selection pressure for VRE, in the absence of both avoparcin and vancomycin usage, could potentially arise from *vanA* being co-selected with other resistance determinants. After the avoparcin ban, the use of anticoccidial ionophore feed additives to control coccidiosis in poultry gradually increased [29]. In Norway, narasin was the anticoccidial ionophore of choice for broilers, though this prophylactic use was phased out in 2015. To the authors’ knowledge, Norway is the only country that has stopped using ionophores as prophylactic feed additive in conventional rearing of broilers. In addition to anticoccidial activity, ionophores have an antibacterial effect [38], mainly against gram-positive bacteria. This has been reflected in frequent isolation of enterococci, especially *E. faecium,* with reduced susceptibility to narasin from broilers in Norway [19,37]. This also include the VRE, as they all display reduced susceptibility to narasin [19]. Nilsson *et al.* showed that resistance to narasin could be transferred between strains of enterococci [13], and later the presence of putative narasin resistance genes were detected on plasmids [39]. These genes were shown to be sufficient to confer narasin resistance and were named *narAB* [14]. Moreover, conjugative co-transfer of narasin and vancomycin resistance [13,14] as well as the co-localization of the *narAB* and the *vanA* genes on plasmids have been reported [39]. However, both Naemi *et al.* [14] and Nilsson *et al.* [13] showed that co-transfer of vancomycin and narasin resistance did not always occur during conjugation, indicating that there may be genetic variations among the isolates. The latter is supported by previous studies, where four narasin- and vancomycin resistance-encoding plasmids (named pVEF1–4) were sequenced, showing a mosaic structure indicative of numerous recombination events [17,18].

In this study we aimed to genetically characterise and compare poultry VRE isolates from Norway and elucidate whether the *narAB* and *vanA* genes were present on the same genetic elements. We also aimed to explore whether co-residence of these genes could be due to predominance of specific VRE clones.

## Materials and methods

### Bacterial isolate collection

Thirty VRE isolates were selected out of 228 VRE isolates from the biobank of the Norwegian surveillance program for antimicrobial resistance in bacteria from animals, food, and feed (NORM-VET) at the Norwegian Veterinary Institute. The selection was not performed to be statistically representative, though selected to some degree to represent different years (2002–2013) and different geographical regions in Norway (Mid, South, East and West). Altogether the selection included 23 broiler isolates and seven turkey isolates (Table 1). Of these, 12 isolates were from the same year (2002), and 5 isolates originated from the same farm. The VRE isolates in the NORM-VET biobank were originally isolated by a selective method. In short, presumptive VRE isolates from broiler flock samples were retrieved from Slanetz and Bartley agar containing 32 mg/L vancomycin, and the species was confirmed by ddlID PCR [40]. Twelve of the isolates (all isolated from 2009 to 2013) were susceptibility tested by the NORM-VET programme (See S1 Table) using the VetMIC™ E-cocci microdilution panels (Swedish National Veterinary Institute, Uppsala Sweden). The antibiotics included in the panel were vancomycin, narasin, kanamycin, tetracycline, linezolid, bacitracin, virginiamycin, erythromycin, ampicillin, gentamicin, streptomycin, and chloramphenicol. Furthermore, 14 of the isolates were previously tested by PCR for the presence of *narAB* [19]*.* Isolates were verified as vancomycin resistant with MIC values above the epidemiological cutoff (ECOFF) value of 4 mg/L, as defined by the European Committee on Antimicrobial Susceptibility Testing (EUCAST). Detailed methodology and information on samples can be found in the annual NORM-VET reports [41].

**Table 1:** Overview of the 29 vancomycin resistant *Enterococcus faecium* isolates included in the study.

**Sequence types (ST)**	ST10 (6), ST195 (3), ST26 (3), ST8 (2), ST248 (2), ST157 (2), ST2713 (2), ST370 (2), ST1866 (1), ST241 (1), ST12 (1), ST1142 (1), ST2511(1), ST2714 (1), ST2715 (1)
**Isolation year**	2002 (13), 2004 (3), 2006 (1), 2007(1), 2009 (1), 2011 (4), 2013 (6)
**Geographical region**	Mid (13), East (11), West (3), South (2)
**Animal host**	Broiler (23), Turkey (6)
**Sample type**	Fecal (27), Environmental (2)

The numbers in parenthesis refer to the number of isolates corresponding to each parameter. For a map of Norwegian geographical regions see S2 Fig.

### DNA extraction

DNA was extracted from LB cultures supplemented with 4 mg/mL vancomycin using QIAamp DNA Mini Kit (Qiagen, Germany) according to the protocol for bacteria in the QIAamp® DNA Mini and Blood Mini Handbook of June 2023 with the modification in that 2500 U/mL Mutanolysin from *Streptomyces globisporus* ATCC 21553 (Sigma Aldrich, US) was used instead of Lysostaphin.

### DNA sequencing

Sequencing libraries from DNA extracts were prepared using the Nextera Illumina DNA Prep kit (Illumina, US). Short-read sequencing of the 30 isolates was performed on an Illumina MiSeq instrument, producing paired-end reads of 300 bp.

A subset of 10 *E. faecium* broiler isolates selected to represent different years and geographical locations of farms were long-read sequenced to obtain contigs of complete chromosomes and plasmids. Extracted DNA was barcoded with the SQK-RBK114.24 ligation sequencing kit and sequenced using a MinION Flow Cell R10.4.1 (Oxford Nanopore Technologies, UK). Basecalling was done with Guppy version 6.5.7 [42] with a minimum quality score of 7 and using the model dna_r10.4.1_e8.2_260bps_sup.cfg.

The raw sequence reads and assemblies are available in the NCBI sequence read archive (SRA) under the BioProject PRJNA1177815 (https://www.ncbi.nlm.nih.gov/bioproject/PRJNA1177815/) with the BioSample accession number SAMN46987761 to SAMN46987790 and SRA Experiment accession number SRX27785198 to SRX27785237.

### Bioinformatic analysis

#### Read assembly.

Short reads from 30 isolates were quality controlled and *de novo* assembled using the Bifrost pipeline [43]. The genome assembly of *E. faecium* ATCC 8459 (GCF_000336405.1) was used as a reference to compare and evaluate the quality and completeness of the newly assembled genomes. In brief, the pipeline runs fastQC version 0.11.9 [44] and MultiQC version 1.9 [45] for quality control, before removing PhiX using BBDuk version 38.76 [46], trimming with Trimmomatic version 0.39 [47] and then assembling with SPAdes version 3.14.0 [48]. Contigs shorter than 500 bp are then removed, before polishing with Pilon version 1.23 [49] using untrimmed reads mapped to the assembly with BWA version 0.7.8 [50], before assembly quality assessment with QUAST version 5.2.0 [51]. See S1 Table for assembly statistics.

Long reads from 10 isolates were demultiplexed with Qcat version 1.1.0 [52] with a minimum read length of 50. Demultiplexed reads were filtered with Nanofilt version 2.8.0 [53] with a minimum quality score of 9 and a minimum read length of 1000. Finally, filtered reads were assembled with Flye version 2.9 [54] with default setting.

Hybrid assemblies were created from short and long reads of ten isolates. In brief, Filtlong version 0.2.1 [55] was used to filter the long reads, discarding the 10% lowest scoring reads, and reads shorter than 1000 bp. These were then combined with the untrimmed Illumina reads and assembled with Unicycler version 0.5.0 [56] in ‘normal’ mode with a minimum contig length of 500 bp and the depth filter set at 0.25. The resulting assemblies were polished with Polypolish version 0.6.0 [57], using a bam file stemming from mapping the raw reads to the assembly with BWA version 0.7.8. See S1 Table for hybrid-assembly statistics.

#### Chromosome and plasmid reconstruction and annotation.

Chromosome and plasmid sequences were reconstructed separately from the 30 draft short read and the 10 hybrid assembly contigs by assigning assembled contigs to either chromosome or plasmid using the MOB-recon tool of MOB-suite version 3.0.3 [58]. Replicons, mating pair formation (MPF) genes and mobilization (MOB) genes were predicted *in silico* by MOB-typer from both short and hybrid assemblies. Only plasmids predicted from hybrid assemblies were circular. All BLAST searches in this study were done with command-line blastn [59] version 2.9.0. The *narAB* genes were identified in reconstructed plasmid contigs from short read assemblies, and in plasmid contigs from hybrid assemblies, by running BLAST with *narAB* (MN590307.1) as query against the contigs (>95% identity and >90% breadth coverage by blastn was considered a positive hit). Reconstructed plasmid sequences from both short and hybrid read assemblies positive for *narAB* were further annotated using Bakta version 1.8.1 with –complete option [60]. A single isolate short read assembly (2006-1402) for which *narAB* and *vanA* were predicted by MOB-suite and ResFinder to reside on different contigs were examined to see if the contigs could possibly be part of the same plasmid. Here, searches with an exemplar IS1216E element from isolate 2011-3991-4 (>95% identity and >70 bp breadth coverage) was used to identify assembly contigs with the reconstructed plasmid-exclusive IS1216E element. A BLAST search for IS1216E elements showed that they in our dataset were exclusive to the reconstructed plasmid contigs, as identified by MOB-suite in hybrid assembled contigs. The predicted size of the resulting plasmid was determined by summing the sizes of all contigs harbouring the IS1216E.

#### Determination of species, sequence types and detection of antimicrobial resistance genes.

The 30 isolates were taxonomically labelled from sequencing reads using Kraken2 version 2.1.2 [61] using default options.

Sequence types (ST) were determined from draft assemblies from short reads using mlst version 2.23.0 [62] with the MLST scheme for *E. faecium* from PubMLST. Four assemblies which initially could not be assigned to known STs were submitted to PubMLST in October 2024 and were assigned novel allele and ST numbers.

Acquired antimicrobial resistance genes (ARGs) and antimicrobial resistance associated with chromosomal point mutations were determined in the reconstructed chromosomes and plasmids from short read and hybrid assemblies using command-line ResFinder version 4.1.0 [63] and PointFinder version 4.1.0 [64], respectively, using default options and databases downloaded May 8th 2023. *OriT* sites were identified by the web-based tool, oriTfinder [65], accessed on 16.06.2024.

#### Pangenome analysis and phylogenies of chromosomes and pVEF plasmids.

Pangenome analysis of reconstructed chromosomes was performed using the “Core gene” track of the ALPPACA pipeline version 2.0.3 [66]. In brief, short read and hybrid assemblies assemblies were annotated using Prokka version 1.14.5 [67] followed by pangenome analysis with Panaroo version 1.2.9. [68].

Reference-based phylogenetic analysis was executed by running the “Mapping” track of ALPPACA pipeline version 2.0.3 [66]. In brief, short reads were mapped to a chosen hybrid assembled reference sequence using Snippy version 4.6.0. [44]. The hybrid chromosome (2004-1570) or plasmid assembly (2011-3991-4) that was used as reference was selected by doing an all against all distance calculation with mash version 2.2.2 [69] selecting the sequence with the lowest average distance to the others as the reference. From the read mappings, Snippy generates a multiple alignment of regions shared by all assemblies (core regions). Recombinant areas in the alignment were identified with Gubbins version 3.3.4 [70] and masked by Maskrc-svg version 0.5 [42]. Finally, constant sites were removed using SNP-sites version 2.5.1. ALPPACA uses IQTree version 2.2.6 [71] to construct a phylogeny from the SNP alignments. The Generalised Time Reversible (GTR) evolutionary model GTR + F + I was used for modelling substitution rates. SNP distances were calculated with snp-dists version 0.8.2 [44]. Phylogenetic trees were visualized in R using the packages ape version 5.7.1 [72], ggtree version 3.8.0 [73], and ggtreeExtra version 1.10.0 [74] for reading, plotting and appending heatmaps to the rectangular trees, respectively. A tanglegram was generated with phytools version 2.3.0 [75]. Coverage and depth were calculated from the bam files using samtools version 1.19.2 [76].

#### Plasmid synteny.

Minimap2 version 2.23 [77] with the options “-X -N 50 -p 0.1 –c” was used to make all-against-all alignments of *narAB*-positive contigs from the long-read hybrid assemblies. The plasmid contigs were indexed using Seqkit version 0.12.0 [78] using the faidx command. Plasmid contigs were compared visually with gggenomes version 1.0.1 [79] in R using the generated GFF3 annotation, index file, and multiple alignment files.

### Ethics statement

This study did not require ethical approval from national committees for medical and health research ethics as the study does not include data or isolates from humans. Poultry isolates were originally collected under the auspices of the national NORM-VET monitoring programme and did not include any animal experiments that required approval from the national animal research authorities, i.e., the Norwegian Food Safety Authorities.

## Results

### Species identification and sequence typing

**Fig 1:**
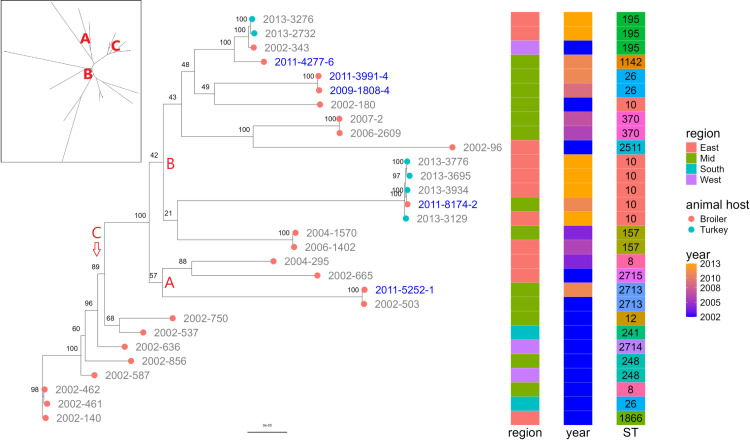
Phylogeny of 29 *Enterococcus faecium* isolates from broiler and turkey flocks sampled in 2002−2013. The tree is based on SNPs from shared regions from short reads mapped to a hybrid assembled reference chromosome from isolate 2004-1570. The rectangular tree is midpoint rooted while the inserted radial tree is unrooted. Isolates with IDs labelled in blue originate from the same farm. A, B, and C represent monophyletic groups.

Of the 30 isolates included, 29 were verified by taxonomic classification as *E. faecium*, while one isolate was re-classified as *E. hirae*. Overall, 15 different STs were detected among the *E. faecium* isolates (Table 1). The 23 broiler isolates belonged to 15 different STs, while the six turkey isolates belonged two STs. The most common ST among all isolates was ST10 (Fig 1). There was a total of six ST10 isolates, including two turkey isolates and four closely related broiler isolates (Fig 1). The *E. hirae* isolate was isolated from chicken feces in the East region of Norway (S2 Fig) in 2002.

### Antimicrobial resistance genes

All the isolates carried the *vanA* and *narAB* genes as anticipated from the selection criteria. In addition, the *ermB* gene*,* which confers resistance to macrolides, was found in one *E. faecium* isolate (2006-2609), while the *tetM* gene, which confers resistance to tetracycline, was found in two *E. faecium* isolates (2002-537 and 2002-750) (S1 Table). The mentioned *narAB*, *vanA*, *ermB*, and *tetM* genes were all found on plasmids in their respective isolates. The intrinsic aminoglycoside resistance gene *aac(6′)-Ii* was detected in all *E. faecium* isolates (S1 Table). A total of 79% (22/29) of the *E. faecium* isolates also carried the intrinsic macrolide resistance gene *msr(C)*. Among the *E. faecium* isolates, 86.2% (25/29) contained mutations in the *pbp5* gene associated with resistance to ampicillin (S1 Table). Two of the turkey isolates and one of the broiler *E. faecium* isolates did not contain these mutations. The *ermB* conferring resistance to macrolides, and the intrinsic resistance gene *aac(6′)-Iid,* were also detected in the *E. hirae* isolate (S1 Table). All intrinsic resistance genes were located on chromosomes.

### Bacterial pangenome and phylogeny

Pangenome analysis showed that the 29 *E. faecium* isolates shared 48% (1871/3881) of the pangenome as core genes, while sharing 49% (1797/3647) of the panchromosome (i.e., excluding plasmid genes.

The phylogenetic analysis was done using SNIPPY, where reads are mapped to a reference. The minimum mapping coverage (the percentage of the reference that an isolate mapped to) among the isolates was 94.2%, and the minimum mapping depth was above 45X for all isolates. The median number of SNPs among the 29 *E. faecium* genomes was 876 and ranged from 10 to 2807. The median number of SNPs among the 23 *E. faecium* isolates from broiler was 808 and ranged from 13 to 2807. Among the six *E. faecium* turkey isolates, the median number of SNPs was 1257 and ranged from 25 to 1285. Phylogeny of the 29 *E. faecium* genomes based on SNPs from shared regions is shown in Fig 1. A monophyletic group A was comprised of four broiler isolates from different years and region. The monophyletic group B was comprised of isolates from different years, regions and from both turkeys and broilers. Within the group B, four turkey isolates from 2013 (i.e., 2013-3776, 2013-3695, 2013-3934 and 2013-3129) and one broiler isolate from 2011 (2011-8174) formed a smaller monophyletic group. The broiler isolate in that smaller group differed by only 10 SNPs from its closest turkey neighbour. A monophyletic group C consisted of broiler isolates all being from the year 2002 but originating from different regions. Only two (2009-1808-4 and 2011-3991-4) of the five isolates originating from the same farm (blue tip labels) demonstrated close phylogenetic relationship. No particular tendencies towards clustering according to geographic region or time was observed for the tree.

### Plasmid characterization and phylogeny

**Fig 2:**
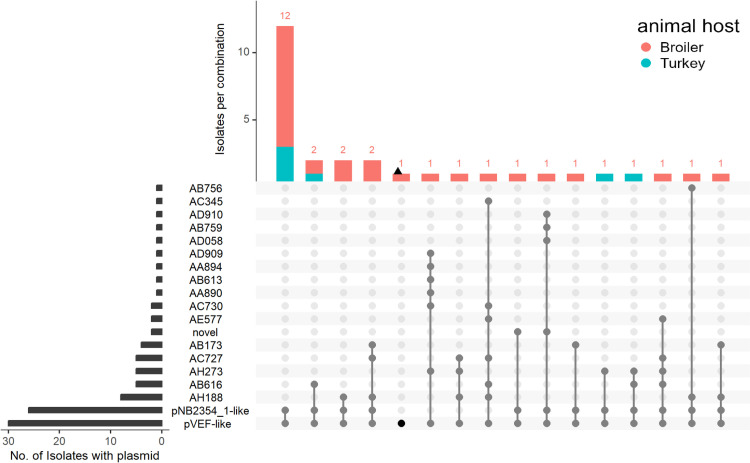
Plasmid content of 29 *Enterococcus faecium* and one *Enterococcus hirae* isolate. The black bars on the left shows the number of isolates with that particular plasmid, while the coloured bars on top shows the number of isolates with that particular combination of plasmids. The grey dots connected by lines in the matrix shows which plasmid combinations are seen. Plasmids are predicted and assigned a cluster ID by MOB-suite. For plasmid content of individual isolates see S1 Table. “novel” refers to predicted plasmids not clustering with any of the plasmids in MOB-suite. The *E. hirae* isolate is represented by a black dot in the matrix and a triangle in the bar.

From the 30 short read assemblies a median of three plasmids was predicted, with a range of one to eight plasmids per isolate assembly. The distribution of these plasmids in the isolates is shown in Fig 2. An overview of the MOB-suite results with the plasmid contents in the individual isolates can be found in S1 Table. The predicted plasmids varied in size from 2.7 kbp to 155 kbp. In total, the plasmids of the study population were assigned to 18 different MOB-suite defined plasmid clusters, of which two smaller plasmids, designated as “novel”, did not cluster with plasmids included in the MOB-suite plasmid database (Fig 2, S1 Table). Overall, 16 different combinations of plasmids were observed in the sequenced isolates.

**Fig 3:**
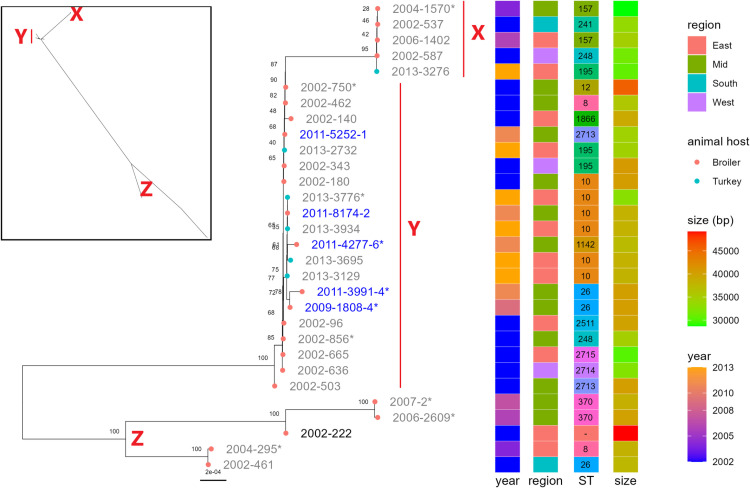
Phylogeny of pVEF-like plasmids from 29 *E. faecium* and one *E. hirae* isolate. The tree is based on core SNPs of short reads from 29 *E. faecium* and one *E. hirae* isolate mapped to the selected reference pVEF-like plasmid from the isolate 2011-3991-4. The rectangular tree is midpoint rooted while the inserted radial tree is unrooted. Isolates labelled in blue originate from the same farm. X and Z are monophyletic groups, while Y is a paraphyletic group. The *E. hirae* isolate is shown with bold label. *Isolates which have been hybrid assembled (Fig 5).

All the isolates carried a plasmid of approximately 40 kbp that contained an average of 45 coding sequences (CDS). These plasmids were assigned to the cluster ID AB172 in the MOB-suite plasmid database and displayed the lowest MASH distance to either of two plasmids, pVEF1 (NC_008768) or pVEF4_A (MG674582), both of which are in the AB172 cluster (S1 Table). Based on the similarity to pVEF1 and pVEF4_A, respectively, we will refer to the AB172 plasmids as pVEF-like plasmids. A phylogeny of our pVEF-like plasmids is shown in Fig 3. The average mapping coverage of the reference was 87.7%, with a low of 46.3% (2006-2609) and a high of 100%. Mapping depth was above 30X for all, except for 2006-2609 which had a depth of 27X. The median SNP distance was 2, with a maximum of 170, and a minimum of 0 SNPs. Most of the pVEF-like plasmids (i.e., 20 of the 29 *E. faecium* plasmids) showed high similarity and formed a paraphyletic group Y with a median number of just 1 SNP. The pVEF-like plasmids in group Y were carried by isolates from both broilers and turkeys, which were originally isolated from different years and different geographical regions. The five pVEF-like plasmids in the monophyletic group X had no SNPs differences and were also carried by both broiler and turkey isolates from different years and geographical regions. The remaining five pVEF-like plasmids in group Z were more diverse with a median number of 31 SNPs. The pVEF-like plasmid carried by the *E. hirae* isolate (2002-222) was among these five.

**Fig 4:**
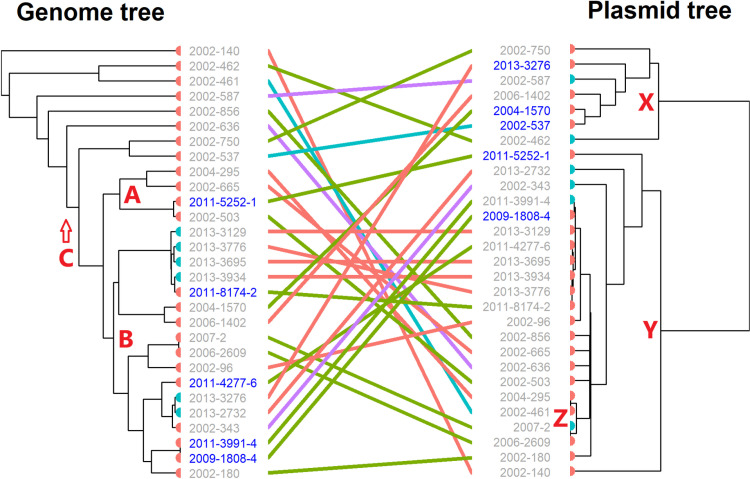
Tanglegram associating the genomes of 29 *Enterococcus faecium* isolates with their corresponding pVEF-like plasmids. The colouring of links between isolate chromosomes and plasmids is according to region. Turkey isolates are represented by blue nodes on the trees. Isolates labelled in blue originates from the same farm.

A tanglegram associating the isolates of the phylogenetic tree of the chromosomes and the pVEF-like plasmids is shown in Fig 4. There is no common phylogenetic topology between the two trees, though some closely related isolates carried closely related pVEF-like plasmids. Examples of this includes both isolates from same geographic location (e.g., 2011-3991-4 and 2009-1808-4) and from different locations (e.g., 2004-1570 and 2006-1402). On the other hand, distantly related isolates were also found to carry related plasmids. Such an example would be that of the pVEF-like plasmids of isolates 2002-461 and 2004-295 differing only by 1 SNP, while the distance between their genomes is 643 SNPs.

A megaplasmid of approximately 100 kbp, showing lowest MASH distance to a plasmid, pNB2354_1 (NC_020208.1), previously identified in *E. faecium* [80], was detected in 89% (26/29) of the *E. faecium* isolates, but not in the *E. hirae* isolate. Like pNB2354_1, this pNB2354_1-like megaplasmid encoded a replication initiator protein of the RepA_N family as well as mobilization genes of the MOBp type, and a transferosome of the MPFt type (S1 Table).

### Composition of *narAB* and *vanA-*encoding pVEF plasmids

**Fig 5:**
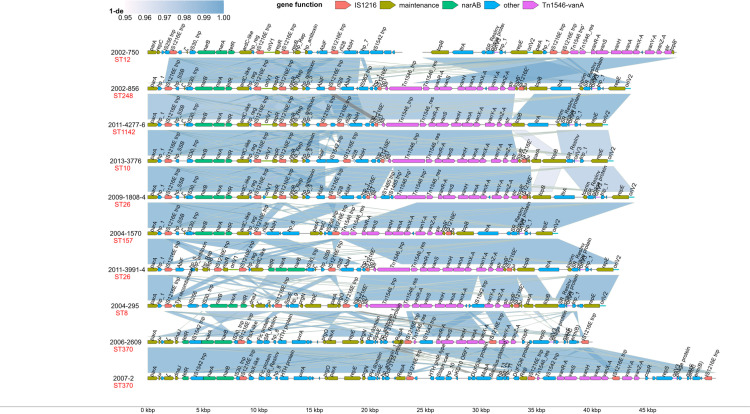
Gene synteny of nine complete and one incomplete pVEF-like-plasmids from vancomycin resistant *Enterococcus faecium* from Norwegian broilers. Genes are colored according to function. The scale legend represents gap-compressed per-base sequence similarity (1 minus the ‘de’ value from minimap2). Gap-compression means that consecutive alignment gaps are counted as one event thereby minimizing the effect of insertion and deletions caused by mobile elements. The 1-de value of 0 should be interpreted as zero matches in alignment, while 1 means only matches. Truncated genes are suffixed by a single quote.

Detection of ARGs in reconstructed plasmids showed that *narAB* and *vanA* genes were predicted to be co-located (i.e., located on the same genetic element) on pVEF-like plasmids in all 29 *E. faecium* isolates, and in the *E. hirae* isolate (S1 Table). Both *narAB* and *vanA* were likewise detected in pVEF1-4 plasmids (AM296544.1, AM410096.1, AM931300.1 and FN424376.1) from Norwegian isolates collected between 1998 and 1999, and previously described to carry *vanA* [17,18,20] From the synteny analysis of the 10 complete hybrid assembled plasmids, it was evident that the *narAB* operon including the *tetR* regulator [14] is intact in all 10 plasmids (Fig 5). The 10 pVEF-like plasmids carried a mean of 3.9 IS1216 elements belonging to the 1216E isoform. In eight of the 10 plasmids, *narAB* was flanked by two convergent (tail-to-tail) IS1216E elements. In the last two plasmids (in isolates 2006-2609 and 2007-2), the two flanking IS1216E elements were in divergent (head-to-head) orientation (Fig 5). The gene order and direction of the resistance-conferring genes of *narAB* and *vanA* operons of the 10 pVEF plasmids were the same among the plasmids. In plasmids from two broiler isolates (2006-2609 and 2007-2), the Tn*1546* transposase gene was absent.

The replication initiation protein, RepE, carrying a PriCT_1 domain typical of the broad-host-range Inc18 plasmid family, was detected on all pVEF-like plasmids (Fig 5 and S1 Table). In eight of the 10 plasmids, a replication origin (oriV2 on Fig 5) was located just upstream from RepE. Six of these eight plasmids additionally carried the replication initiator protein, RepR, belonging to the Rep_3 family, and a neighbouring replication origin (oriV1) (Fig 5). The two plasmids in which repE was not associated with an oriV2 site (2006-2609 and 2007-2) instead carried a different PriCT_1 family replication initiator protein, repS, and a neighbouring oriV3 site. Additionally, these two plasmids carried RepA of the Rep_trans family, with the plasmid of 2007-2 further carrying Rep belonging to the Rep_3 family.

In six of the 10 circular pVEF plasmids, an *oriT_pC221* of 53 bp was located between a truncated IS1216E transposase and a truncated streptomycin resistance gene (*str*) (Fig 5). The *oriT* site (origin of transfer) is a specific DNA sequence in bacterial plasmids from where the process of conjugative DNA transfer begins. However, no genes encoding a conjugation machinery were identified (S1 Table).

Three of the 10 circular pVEF plasmids (2004-295, 2006-2609 and 2007-2) encoded intact omega-epsilon-zeta (ω-ε-ζ) toxin-antitoxin (TA) as previously described for pVEF1-3 [17,18]. These three plasmids all belonged to the monophyletic group Z in the phylogenetic tree of plasmids (Fig 3). The remainder seven plasmids, all belonging to monophyletic groups X and Y, were either not encoding the zeta toxin (2013-3776), or encoding a truncated toxin as previously described for pVEF4 [20]. In our plasmids, the truncated toxin contained only the 116 bp C-terminal region of the full 863 bp toxin, excluding the N-terminal necessary for toxicity and plasmid maintenance [81].

## Discussion

This is the first study using whole genome sequencing to genetically characterize vancomycin and narasin resistant enterococci isolated from Norwegian broilers and turkey. Whole genome sequencing data showed high genetic variability among the VRE isolates, and that for all the analyzed isolates, the narasin and vancomycin resistance determinants were co-localized on pVEF-like plasmids. The *E. faecium* poultry isolates shared a higher number of core genes and had smaller pangenome and pan-chromosome than recently described for 28 human clinical VRE isolates, collected during 2018 and 2019 from an Irish hospital [82], which shared only 19% (1324/7118) and 23% (1327/5868) of the pangenome and pan-chromosome, respectively [82]. However, phylogenetic analysis and MLST demonstrated high genetic variability within the population. A heterogeneous VRE population in Norwegian broilers has been described previously, at the time investigated by pulsed-field gel electrophoresis [9,83]. This also aligns with a generally recognized high genome plasticity for *E. faecium* which has been suggested to be driven by the presence of mobile elements such as insertion sequences [84]. The high genetic variability among the isolates indicates that co-occurrence of *vanA* and *narAB* on pVEF-like plasmids in Norwegian broiler isolates is not due to clonal dissemination. This is in contrast to results to Sweden, where the dominant ST310 was hypothesized to be responsible for a rise of VRE in Swedish broilers from 2001 to 2020 [13,85,86]. ST310 was not detected in the present study. However, three of the isolates in our study (sequence typed as ST8, ST248, and ST370) do belong to STs also detected in Swedish VRE isolates from broilers collected between 2000 and 2009 [13]. The Norwegian broiler production system follows a pyramidal structure depending on import of eggs from Sweden. These imported eggs are used to produce the parent stock, whose day-old chickens are distributed to broiler farms across the country. Consequently, broilers in Norway and Sweden have a common origin. This makes vertical transmission of the same VRE STs downwards in the production pyramid in both countries feasible, as previously seen for quinolone resistant *Escherichia coli* [87,88] and for *E. coli* resistant to extended spectrum cephalosporins [89–91]. Failure to detect ST310 among the isolates in Norway could be explained by persistence of the clone in the environment of Swedish broiler farms [92], and potential absence of the clone in layer environments. However, it should be noted that the present study consists of relatively few isolates, and we cannot exclude that VRE ST310 isolates have been present at Norwegian broiler farms.

One of the broiler isolates (2011-8174-1) clustered together with four turkey isolates, suggesting a potential relationship between these VRE isolates across different poultry host species. However, as the turkey isolates are from 2013 and the broiler isolate is from 2011, and they originate from farms in different regions, there is no obvious epidemiological link. In addition, one of the pVEF plasmids in the study was found in *E. hirae*, suggesting that the pVEF-like plasmids can spread between bacterial species.

The *vanA* genes have previously been found in Norwegian and Danish *E. faecium* broiler isolates, on plasmids designated as pVEF1-4 [17,18,20] and pVEF4_A [93], respectively. These plasmids share features with the pVEF-like plasmids characterized in this study. All these plasmids display mosaic structures with multiple replicons [17,18,20]. We furthermore found all these plasmids to be carrying the *narAB-*operon. The *vanA* gene cluster is embedded in a non-conjugative simple transposon Tn*1546*, which is capable of replicative transposition [20,94]. This Tn*1546* transposon is spatially separated from the *narAB* genes in all pVEF plasmids that have been characterized so far [17,18,20]. The pVEF-like plasmids investigated in the present study are relatively conserved in the core regions used for SNP analysis and thereby appear similar in the phylogenetic analysis. Additionally, gene synteny analysis of the functional segments of the plasmids reveal considerable similarity in the structure of all the pVEF-like plasmids. The functional segments of the plasmids are interspersed with IS1216E insertion sequences from the IS6/IS26 family. Intact IS1216E elements are flanking a unit that contains *narAB*. In eight of the 10 pVEF-like complete plasmids, the IS1216E elements are in convergent orientation. IS1216-based composite transposons carrying antimicrobial resistance genes have recently been reported in *E. faecium* [95,96]. It is therefore likely that *narAB* flanked by IS1216E elements constitute a composite transposon. The order of the genes in the regions between *narAB* and *vanA,* which span more than 10 kb, is not conserved between the characterized plasmids, which implies that these resistance mechanisms are likely not transferred as a single transposable unit. However, it should be noted that they are still being maintained on the same plasmid and could be co-transferred by conjugation.

The persistence of VRE in poultry in the absence of an obvious selection pressure for vancomycin resistance has been debated in scientific literature [14,18,39]. VRE from Nordic poultry have been demonstrated to persist in the environment of broiler farms despite the practice of an ‘all-in, all-out’ strategy with cleaning, disinfection, and downtime between flocks [23,92,97]. However, the eventual reduction of VRE in Norwegian broiler farms after decades of persistence suggests that undescribed selection pressures exist in these environments. It has been hypothesized that the presence of the three-component omega-epsilon-zeta TA-system in pVEF1-3 might contribute to the long-term maintenance of resistance plasmids [18]. However, in both pVEF4 [20] and in seven of the 10 complete pVEF-like plasmids in this study, the zeta toxin was truncated. In only three pVEF-like plasmids (2004-295, 2006-2609 and 2007-2) the TA-system was intact. This indicates that the omega-epsilon-zeta TA-system is in general not responsible for persistence of these plasmids in enterococci of Norwegian poultry.

Conjugative transfer of plasmids requires the presence of *oriT* and the action of a conjugation machinery consisting of two sets of genes, i.e., mating pair formation (MPF) genes and mobilization (MOB) genes [98]. Plasmids encoding an *oriT* and a conjugation machinery are called conjugative plasmids, while plasmids encoding *oriT*, but requiring MOB and/or MPF *in trans* from a helper plasmid, are termed mobilizable plasmids [98]. While an *oriT_pC221* site was identified in six of 10 complete pVEF-like plasmids in this study, none of them encoded a conjugation machinery. The *oriT_pC221* site was originally described for the chloramphenicol resistance conferring pC221 plasmids isolated from *Staphylococcus aureus* [99]. Conjugation of plasmid pC221 is dependent on MOBp type genes supplied in trans, indicating that this might also be the case for the pVEF-like plasmid. Indeed, MOBp and MPF genes were found on the pNB2354-1-like megaplasmids of approximately 100 kbp detected in 26 of the 29 *E. faecium* isolates. The finding of megaplasmids is not reported in the earlier plasmid analysis of Norwegian VRE isolates [17,18,20]. However, Swedish broiler isolates collected in 2000 and 2007 [13], and Danish broiler isolates collected in 2010 [100] and 2016 [93] were likewise noted to carry megaplasmids. The megaplasmids of three Danish VRE isolated from retail chicken meat in 2016 also encoded replication initiators belonging to RepA_N [93]. Taken together, these findings suggests that the pNB2354-1-like megaplasmid could serve as a helper plasmid for mobilization of the pVEF-like plasmids by providing MPF and MOB genes in trans. Further studies are needed to confirm the potential role of this megaplasmid with regard to plasmid conjugation.

The synteny plot reveals that a majority of genetic features on the hybrid assembled pVEF-like plasmids can be assigned to either mobile genetic elements (mainly IS1216E but also IS30, IS256 and the group II intron, LtrA), plasmid maintenance functions such as replication, partitioning, and anti-restriction genes (*ardC*), or antimicrobial resistance genes (*narAB* and *vanA*). Other features are the *abiF* and *abiH* genes encoding two different phage abortive infection (Abi) systems that were carried on four and seven of the 10 pVEF-like plasmids, respectively. While Abi is a broad term for anti-phage systems which inhibit phage replication after DNA entry, the mechanism of *abiF* and *abiH* are currently unknown [101]. The absence of functions such as virulence, nutrient utilization, conjugation, or antibacterial activities suggests that the main function of pVEF-like plasmids is related to resistance mediated by *narAB* and *vanA*.

The pVEF-like plasmids in this study demonstrated diversity in gene order, an abundance of IS elements, and strong conservation of shared gene core regions, which together suggests that the plasmids undergo genetic re-arrangement. However, highly similar plasmids originating from genetically diverse isolates indicate that these plasmids may also be transferred between isolates. The pVEF-like plasmids in this study were broad-host-range Inc18 plasmids that have been detected in enterococci, streptococci and staphylococci, indicating the possibility of interspecies transfer [102]. This is supported by the carriage in this study of the pVEF-like plasmid in *E. hirae*. There is a concern that these plasmids could be transferred from animal to human pathogenic enterococci and other important human pathogens such as MRSA. Conjugative transfer of pVEF1 and pVEF2 from poultry isolates to a human commensal *E. faecium* has previously been demonstrated [11]. Moreover, in vitro transfer of *vanA*-encoding plasmids from *E. faecium* isolates to *S. aureus* has been shown [16].

Considering the potential for transfer of these Inc18 plasmids from animal host bacteria to humans, the presence of this *vanA* containing pVEF plasmid in VRE from poultry is concerning. Such occurrence of VRE in animal populations is a risk factor for transfer to the community and to healthcare settings that should not be neglected. It was recently reported from Taiwan that there has been a continuous increase in clinical VRE infections from 2004 to 2018 [103]. The proportion of the VREs carrying the *narAB* genes increased from 4% in 2010 to 39% in 2018 likely due to clonal expansion of an ST17 strain. The occurrence of VRE carrying *narAB* genes strongly suggests dissemination of bacteria or plasmids from animals to humans and either horizontal gene transfer of the plasmid to a human adapted strain or the adaptation of VRE of animal origin to the human host. The occurrence of VRE in animal populations is not monitored routinely and is likely severely underreported globally. The risk of dissemination of VRE from animals to the community likely differs between countries depending on culture and tradition in terms of animal production and frequency of human and animal interactions.

## Conclusion

To conclude, the results in the present study support that vancomycin and narasin resistance are co-located and co-selected on pVEF-like plasmids in Norwegian broiler and turkey VRE isolates. The pVEF-like plasmids are found in genetically diverse backgrounds, i.e., *E. faecium* of several different STs and in an *E. hirae* isolate and appear to be dedicated to the function of transferring narasin and vancomycin resistance. Thereby, use of ionophores like narasin as feed additives to control coccidiosis probably have contributed to maintaining a VRE reservoir after the avoparcin ban. Moreover, the results indicate that interspecies plasmid transfer, i.e., between *E. faecium* and *E. hirae*, may occur, and that a commonly co-resident megaplasmid probably act as a helper plasmid for such transfer. This study is however limited by its modest sample size, and a more thorough study on a larger number of VRE isolates is needed to follow up on the results in this study.

## Supporting information

S1 Table Stats, results, and metadata from contig assemblies of 30 VRE isolates. The median N50 value of the short-read assemblies was 41.15 kbp with a range from 29.4 to 386.3, and median genome size was 2.5 Mbp with a range from 2.5 to 3.1. The median N50 value of the hybrid assemblies was 2.47 Mbp with a range from 2.41 to 2.59, and the median number of contigs was 4.5 with a range from 2 to 11. The sum was of zero indicates that all assemblies were complete.
(XLSX)

S1 Fig Genetic variability of 29 vancomycin resistant *Enterococcus faecium* poultry isolates based on SNP-distances between 1797 core genes. Turkey isolates are indicated by red fonts and broiler isolates by black.
(TIF)

S2 Fig Map of Norwegian geographic regions. Map is divided into the regions North (Northern Norway), Mid (Trøndelag), West (Western Norway) and South (Southern Norway). The geospatial data was downloaded from Norway’s national geospatial data portal and is unofficial and suitable for illustrative purposes. The figure complies with the CC BY 4.0 license.
(TIF)
