# Supplementary figures and images for: Genomic characterization of vancomycin-resistant enterococci in Norwegian poultry
Source: PLoS One. 2025 Jun 4;20(6):e0324789. doi: 10.1371/journal.pone.0324789 (PMC12136351; doi:10.1371/journal.pone.0324789)

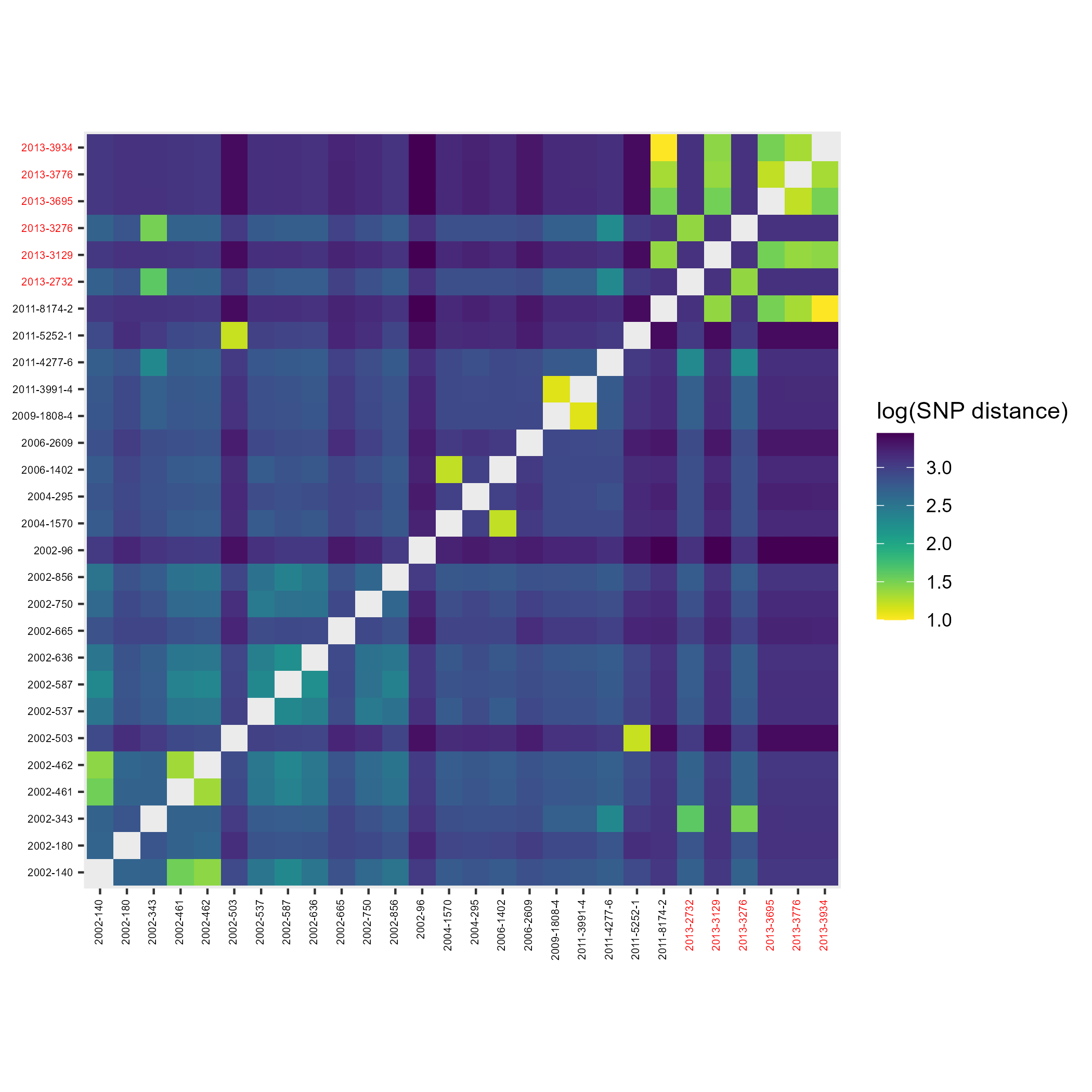

Supplement: S1 Fig — Turkey isolates are indicated by red fonts and broiler isolates by black. (TIF) [file pone.0324789.s002.tif]

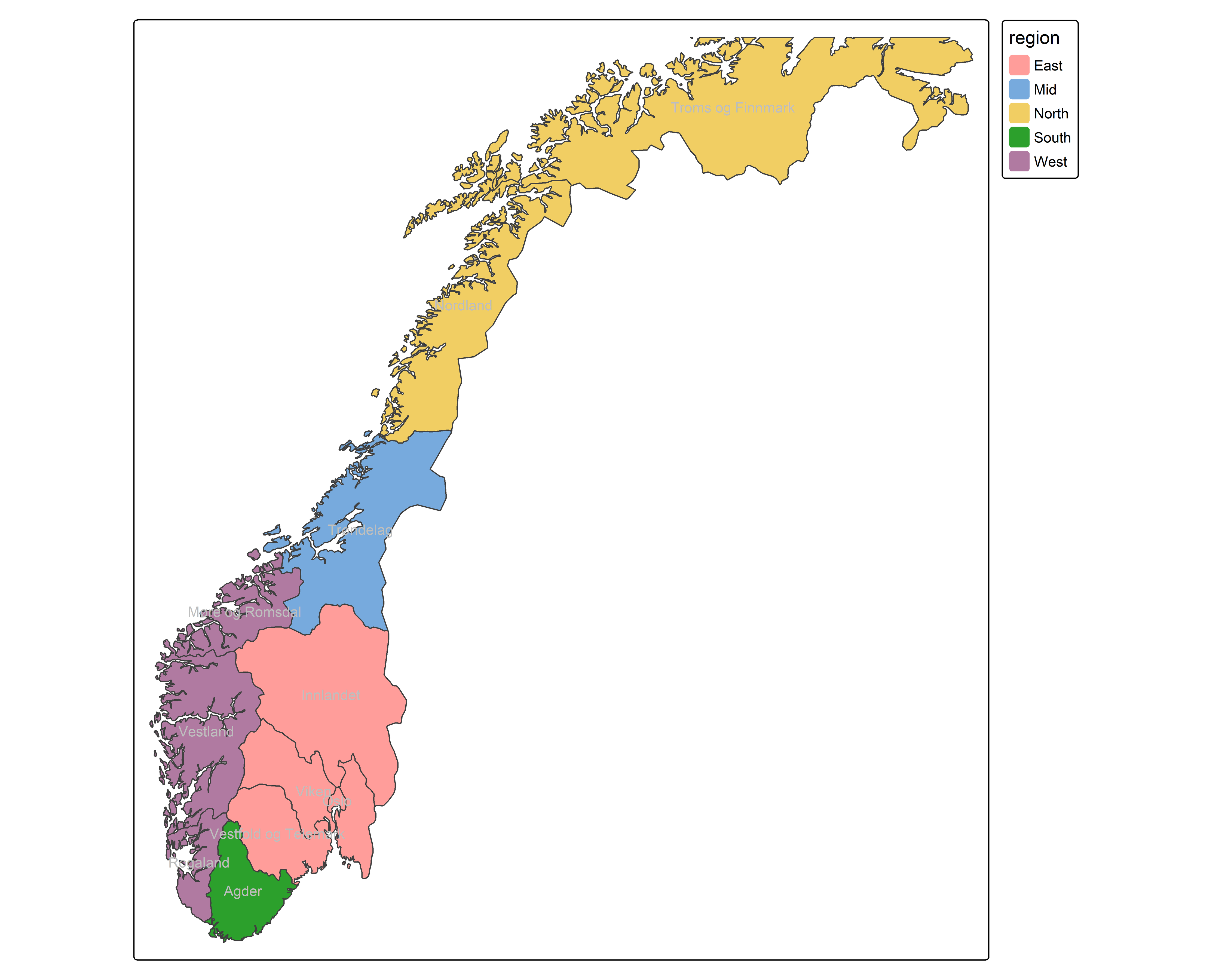

Supplement: S2 Fig — Map is divided into the regions North (Northern Norway), Mid (Trøndelag), West (Western Norway) and South (Southern Norway). The geospatial data was downloaded from Norway’s national geospatial data portal and is unofficial and suitable for illustrative purposes. The figure complies with the CC BY 4.0 license. (TIF) [file pone.0324789.s003.tif]
